# Sex Differences in Stroke Subtypes, Severity, Risk Factors, and Outcomes among Elderly Patients with Acute Ischemic Stroke

**DOI:** 10.3389/fnagi.2015.00174

**Published:** 2015-09-08

**Authors:** Changshen Yu, Zhongping An, Wenjuan Zhao, Wanjun Wang, Chunlin Gao, Shoufeng Liu, Jinghua Wang, Jialing Wu

**Affiliations:** ^1^Department of Neurology, Tianjin Huanhu Hospital, Tianjin, China; ^2^Key Laboratory of Cerebral Vascular Disease and Neurodegenerative Disease, Tianjin, China; ^3^Department of Epidemiology, Tianjin Neurological Institute, Tianjin, China

**Keywords:** ischemic stroke, sex differences, risk factors, outcomes, elderly

## Abstract

**Background:**

Although the age-specific incidence and mortality of stroke is higher among men, stroke has a greater clinical effect on women. However, the sex differences in stroke among elderly patients are unknown. Therefore, we aimed to assess the sex differences in stroke among elderly stroke patients.

**Methods:**

Between 2005 and 2013, we recruited 1484 consecutive acute ischemic stroke (AIS) patients (≥75 years old) from a specialized neurology hospital in Tianjin, China. Information regarding their stroke subtypes, severity, risk factors, and outcomes at 3 and 12 months after stroke were recorded.

**Results:**

Comparing with men, women had a significantly higher prevalence of severe stroke (17.20 vs. 12.54%), hypertension (76.42 vs. 66.39%), dyslipidemias (30.35 vs. 22.76%), and obesity (18.40 vs. 9.32%), *P* < 0.05. Comparing with women, men had a significantly higher prevalence of intracranial artery stenosis (23.11 vs. 17.45%), current smoking (29.60 vs. 13.05%), and alcohol consumption (12.15 vs. 0.47%), *P* < 0.05. Moreover, dependency was more common among women at 3 and 12 months after stroke, although the sex difference disappeared after adjusting for stroke subtypes, severity, and risk factors.

**Conclusion:**

Elderly women with AIS had more severe stroke status and worse outcomes at 3 and 12 months after stroke. Thus, elderly female post-AIS patients are a crucial population that should be assisted with controlling their risk factors for stroke and changing their lifestyle.

## Introduction

The latest report indicates that stroke was the second most common cause of death and the third most common cause of reduced disability-adjusted life-years worldwide in 2010 (GBD 2013 Mortality and Causes of Death Collaborators, 2015). Unfortunately, stroke has a greater clinical effect on women than on men (Kelly et al., 2003; Di Carlo et al., 2003; Kapral et al., 2005). Previous studies have also reported that the underlying etiology, causes, and burden of stroke may be different for each sex, and that differences in men’s and women’s physiology may affect the sex differences in stroke. Although the age-specific stroke incidence and mortality is higher among men, the total number of strokes is higher and the outcomes are worse among women, as women live longer and the risk of stroke increases with age (Niewada et al., 2005; Reeves et al., 2008). Unfortunately, this difference will become increasingly significant in the coming decades. The Chinese population is also aging, with 200 million residents who were ≥65 years old in 2014, and 3.5% of the population ≥75 years old in 2013 (Peilin et al., 2014; National Bureaus of Statistics of the People’s Republic of China, 2014). Nevertheless, few studies have documented the sex differences of stroke among elderly stroke patients.

In this study, we evaluated patient information using a hospital-based stroke registry from Tianjin, northern China. To the best of our knowledge, this is the first prospective study that has aimed to assess the sex differences in pre-stroke risk factors, severity, and 3- and 12-month outcomes among elderly patients in northern China.

## Materials and Methods

All patients were from a stroke registry established in 2005 in Tianjin Huanhu Hospital. Between January 2005 and December 2013, we recruited consecutive patients aged ≥75 years old with first-ever acute ischemic stroke (AIS) who were admitted to the stroke unit of Tianjin Huanhu Hospital, which is a specialized neurology hospital in Tianjin, China. A clinical diagnosis of stroke was made according to the World Health Organization’s criteria, and all diagnoses were confirmed using brain computed tomography or magnetic resonance imaging (World Health Organization Task Force on Stroke and Other Cerebrovascular Disorders, 1989). Patients with transient ischemic attack were excluded from this study.

The study was approved by the ethics committee for medical research at Tianjin Huanhu Hospital and the Tianjin Health Bureau, and a written informed consent for each participant was obtained.

Among AIS patients, detailed information was obtained from the stroke registry system of Tianjin Huanhu Hospital, which evaluated the stroke subtypes, stroke severity, previous medical history, stroke risk factors, and outcomes at 3 and 12 months after stroke.

The stroke subtypes were classified according to the Trial of Org 10172 in Acute Stroke Treatment (TOAST) criteria, and were defined as atherothrombotic, cardioembolic, lacunar, other causes, and undetermined (Adams et al., 1993). The patient’s neurological function deficit was evaluated using the National Institute of Health stroke scale (NIHSS), Bethel index (BI), and the modified rank scale (mRS) on admission. Stroke severity was categorized using the NIHSS into three groups: mild (NIHSS: ≤7), moderate (NIHSS: 8–16), and severe (NIHSS: ≥17) (Bamford et al., 1991).

The stroke risk factors included the medical history of hypertension, diabetes mellitus (DM), dyslipidemias, atrial fibrillation (AF), and intracranial artery stenosis. We also evaluated modifiable lifestyle factors, including current smoking (≥1 cigarette per day for ≥1 year), alcohol consumption (≥1 drink per week for 1 year), and obesity [body mass index (BMI) ≥30 kg/m^2^].

The patient outcomes included mortality, reoccurrence, and dependency rate at 3 and 12 months after stroke. Death was defined as all-cause cumulative death at the corresponding time points after stroke. Recurrence was defined as the proportion of patients with all new-onset vascular events (stroke, myocardial infarction, and venous thrombosis) after 30 days of initial stroke in all survivors’ patients. Dependency was defined as the proportion of patients with mRS >2 among all survivors (Ji-Sun et al., 2010).

This study was carried out in accordance with the ethics committee of Tianjin Huanhu Hospital with written informed consent from all subjects. All subjects gave written informed consent in accordance with the Declaration of Helsinki.

Descriptive statistics were used to compare the sex differences. The continuous variables were presented as mean (SD) or median (range), and were compared using the *t* test or Mann–Whitney *U* test, as appropriate. Dichotomous variables were presented as numbers (percentages), and were compared using the chi-square test. The sex differences in outcomes were assessed using logistic regression models, and the risk was presented using unadjusted odds ratios (OR) and 95% confidence intervals (CI). The multivariate analysis was carried out using a logistic regression model with stroke subtypes, stroke severity, and risk factors as the covariates; the results were presented using the adjusted OR with 95% CI. All statistical analyses were performed using SPSS version 15.0 (SPSS Inc., Chicago, IL, USA), and *P* < 0.05 for two-tailed tests was used to determine statistical significance.

## Results

**Figure 1:**
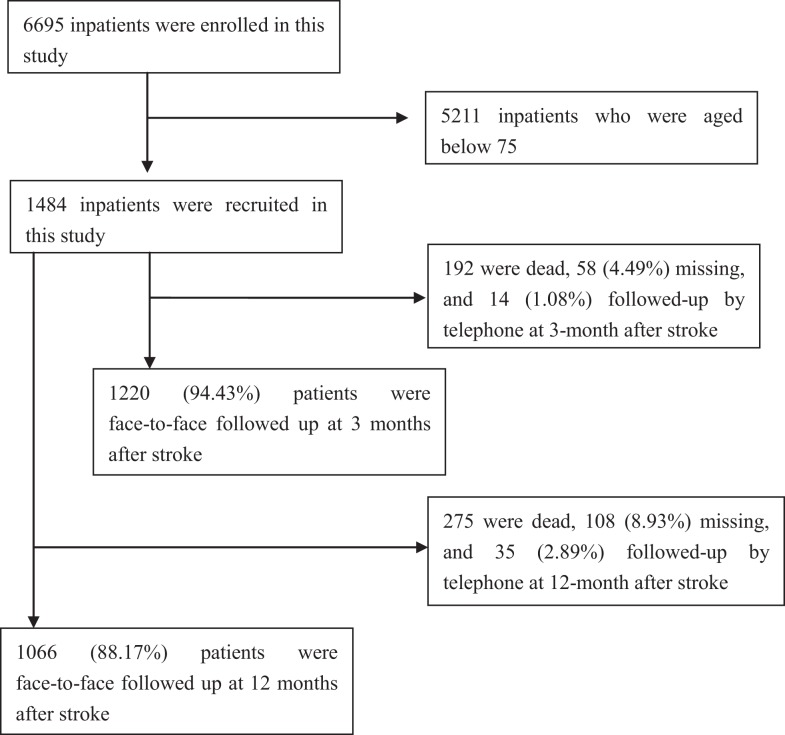
**Flow diagram of participants**.

A total of 6695 patients with first-ever AIS were recruited during the study period, 1484 patients were included in this study after excluding 5211 patients aged <75 years. Of these patients, the mean age of stroke onset was 79.63 ± 4.00 years (from 75 to 96) overall; 79.69 ± 3.99 years (from 75 to 96) in men, and 79.55 ± 4.01 years (from 75 to 95) in women. The responding rate was 94.43% at 3 months after AIS, and 88.17% at 12 months after AIS (Figure 1).

Among the 1484 consecutive AIS patients, 848 patients (57.1%) were men and 636 patients (42.9%) were women. Table 1 showed that TOAST classifications were similar for men and women. The median NIHSS and BI on admission were higher for women than for men, although the mRS was similar for both sexes.

**Table 1:** **The clinical characteristics of stroke subtypes and severity in ischemic stroke patients aged 75 years and over**.

Characteristics	Men	Women	*P*
Numbers, *n* (%)	848 (57.14)	636 (42.86)	–
TOAST classification			0.513
Atherothrombotic	535 (63.09)	383 (60.22)	
Small-artery disease	165 (19.46)	125 (19.65)	
Cardiac embolism	86 (10.14)	83 (13.05)	
Others determined etiology	6 (0.71)	5 (0.79)	
Undetermined etiology	56 (6.60)	40 (6.29)	
Stroke severity			0.009
Mild	513 (61.29)	340 (54.14)	
Moderate	219 (26.16)	180 (28.66)	
Severe	105 (12.54)	108 (17.20)	
Neurological function deficit			
NIHSS	6.35 (0–36)	7.75 (0–37)	0.001
BI	50.44 (0–100)	43.55 (0–100)	<0.001
mRS	3.82 (0–6)	3.99 (0–6)	0.228

The prevalence of hypertension, dyslipidemias, and obesity among elder women with AIS were 76.42, 30.35, and 18.4%, respectively, and the corresponding prevalence in elderly men with AIS were 66.39, 22.76, and 9.32%, respectively. A greater prevalence of hypertension, dyslipidemia, and obesity was observed among women in this study. By contrast, women were less likely to have a history of intracranial artery stenosis, current smoking, and alcohol consumption compared to men (17.45 vs. 23.11, 13.05 vs. 29.6, and 0.47 vs. 12.15%, respectively; *P* < 0.05). However, no sex differences were observed in the prevalence of DM, and AF, *P* > 0.05 (Table 2).

**Table 2:** **Sex differences in risk factors of stroke in patients aged 75 years and over**.

Risk factors	Men	Women	*P*
Hypertension	563 (66.39)	486 (76.42)	<0.001
Diabetes	224 (26.42)	191 (30.03)	0.125
Atrial fibrillation	137 (16.16)	120 (18.87)	0.172
Dyslipidemias	193 (22.76)	193 (30.35)	0.001
Obesity	79 (9.32)	117 (18.40)	<0.001
Intracranial artery stenosis	196 (23.11)	111 (17.45)	0.008
Current smoking	251 (29.60)	83 (13.05)	<0.001
Alcohol consumption	103 (12.15)	3 (0.47)	<0.001

No significant sex differences were observed in the mortality and recurrence rates at 3 and 12 months after stroke (Table 3). There were significantly higher dependency rates in women than those in men, 45.39 vs. 38.33% at 3 months, and 33.89 vs. 26.33% at 12 months *P* < 0.05. The RR (95% CI) of dependency in women was 1.34 (1.04–1.72, *P* = 0.024) at 3 months, and 1.43 (1.00–2.07, *P* = 0.050) at 12 months, comparing to men. However, the sex difference disappeared after adjusting for stroke subtypes, severity, and risk factors.

**Table 3:** **Sex differences in outcome after stroke in 3 and 12 months among patients with ischemic stroke aged 75 years and over**.

Outcomes	Men	Women	Unadjusted		Adjusted	
			OR (95%CI)	*P*	OR (95%CI)	*P*
3 months
Mortality	117 (14.16)	75 (12.48)	0.94 (0.83, 1.07)	0.357	0.70 (0.48, 1.04)	0.074
Dependency	225 (38.33)	197 (45.39)	1.34 (1.04, 1. 72)	0.024	1.23 (0.89, 1.71)	0.206
Recurrence	122 (14.91)	86 (14.45)	0.99 (0.87, 1.02)	0.809	0.89 (0.64, 1.25)	0.506
12 months
Mortality	164 (20.53)	111 (19.27)	0.97 (0.87, 1.08)	0.566	0.76 (0.55, 1.06)	0.108
Dependency	84 (26.33)	81 (33.89)	1.43 (1.00, 2.07)	0.050	1.25 (0.77, 2.04)	0.375
Recurrence	247 (31.79)	173 (30.84)	0.98 (0.89, 1.08)	0.711	0.88 (0.68, 1.14)	0.335

Moreover, Table 4 presented that the dependency rate was only associated with AF at 3 months after stroke, with the dependency rate of 50.3% in patients with AF, and 39.8% in patients without AF, *P* = 0.0174, no significant associations were found in other stroke risk factors, all *P* > 0.05. However, there were trends of increased dependency rate with stroke severity at 3 and 12 months after stroke, with the dependency rate of 22.3% in mild patients, 78.1% in moderate patients, and 97.1% in severe patients at 3 months; the corresponding rate at 12 months after stroke was 12.5, 68.7, and 95.8%, respectively, *P* < 0.001.

**Table 4:** **The association between dependency rate and stroke risk factors in patients aged 75 years and over**.

Risk factors	Without risk factors	With risk factors	*P*
3 months
Hypertension	114 (38.6)	308 (42.4)	0.266
Diabetes	293 (40.0)	129 (44.6)	0.178
Atrial fibrillation	349 (39.8)	73 (50.3)	0.017
Dyslipidemias	306 (41.2)	116 (41.6)	0.922
Obesity	351 (40.2)	71 (48.0)	0.076
Intracranial artery stenosis	324 (40.9)	98 (42.8)	0.610
Current smoking	328 (41.7)	94 (40.2)	0.681
Alcohol consumption	396 (41.7)	26 (36.1)	0.351
12 months
Hypertension	40 (24.7)	125 (31.6)	0.106
Diabetes	113 (28.8)	52 (31.3)	0.554
Atrial fibrillation	139 (28.4)	26 (38.2)	0.095
Dyslipidemias	123 (30.4)	116 (41.6)	0.922
Obesity	135 (28.2)	30 (37.5)	0.093
Intracranial artery stenosis	129 (29.6)	98 (42.8)	0.610
Current smoking	132 (30.6)	33 (26.0)	0.314
Alcohol consumption	154 (29.7)	11 (28.2)	0.846

## Discussion

Women have a higher lifetime risk of stroke than men, and the stroke mortality is higher among women than among men in high-income countries. The stroke prevalence is expected to increase among older women (Banks and Marotta, 2007). These facts are often attributed to the longer life expectancy of women. In this study, we assessed the sex differences in stroke subtypes, severity, risk factors, and outcomes 3 and 12 months after stroke among AIS patients who were ≥75 years old.

Regarding the TOAST criteria, previous studies have reported that women are more likely to have a cardioembolic stroke, and that men are more likely to have a large or small vessel stroke (Valery et al., 2014; Gray et al., 2007; Eileen et al., 2009). By contrast, another study found no sex difference in the TOAST classifications (Smith et al., 2005). Similarly, we found that cardioembolic stroke accounted for 13.1% of all stokes among women (vs. 10.1% among men), although this difference was not statistically significant. This rate is lower than the latest report, which the proportion of cardioembolic stroke was 19% (Mehndiratta et al., 2015). Interestingly, the proportion of large-artery atherosclerosis in present study was >60%, which is more than that reported in other studies. One major reason for this difference is that our study used a hospital registry, patients were more likely to be severe. A second reason may be related to the use of radiology, as cardioembolisms and small-artery occlusion may be considered large-artery atheroscleroses. Finally, our patients were ≥75 years old, and may have different stoke etiologies and pathologies compared to younger patients.

A number of studies have evaluated the sex difference in stroke severity, and several have confirmed that women have increased severity stroke compared to men, as measured using NIHSS at admission (Lai et al., 2005; Wesley et al., 2010; Kevin et al., 2007). By contrast, another study evaluated the first-ever ischemic stroke via NIHSS, and reported no sex difference (Olsen et al., 2007). However, few studies have addressed this difference among elderly AIS patients. In the present study, we observed that women had significantly higher median NIHSS and BI scores at admission, and were significantly more likely to have a severe stroke, compared to men. This result indicates that a sex difference in stroke severity exists among elderly Chinese AIS patients, and this finding may be partially explained by the slightly increased prevalence of cardioembolic stroke among women. Women were less likely to present to a hospital within 3 h as compared to men (Mehndiratta et al., 2015), which may contribute to severe stroke situation on admission in women.

The sex differences regarding traditional stroke risk factors have also been reported in previous studies. Hypertension, DM, AF, and high cholesterol level are all important modifiable stroke risk factors, while smoking, alcohol consumption, and obesity are all important modifiable lifestyle risk factors (Goldstein et al., 2006). In addition, recent studies have reported that women are more likely to have hypertension, DM, AF, and obesity, whereas men are more likely to have a history of heart disease, myocardial infarction, peripheral arterial disease, current smoking, and alcohol consumption (Reeves et al., 2008; Kapral et al., 2005; Klaus et al., 2010; Gall et al., 2010). Furthermore, recent data also suggest that women are more likely to have a family history of stroke (Touze and Rothwell, 2007; Touze and Rothwell, 2008). Finally, elderly women are more likely to have DM, hypertension, dyslipidemia, and heart disease than elderly men (Yuehua et al., 2013). In this study, we found that elderly women with AIS were more likely to have hypertension, dyslipidemias, and obesity, and men were more likely to have a history of intracranial artery stenosis, current smoking, and alcohol consumption. However, no differences were observed regarding the prevalence of DM and AF. In addition, we found that women had higher levels of TC, TG, HDL-C, and LDL-C. These finding indicate that elderly women were more likely to develop abnormal lipid metabolism compared to men.

The outcomes after stroke among elderly women have become a global focus point in this field. Previous studies have reported that women have worse functional outcomes after stroke, although there were no sex difference after adjusting for the various confounding factors (such as age and stroke severity) (Ji-Sun et al., 2010; West et al., 2010; Kong et al., 2010; Vibo et al., 2007; Shen et al., 2006; Ní Chróinín et al., 2011; Dhamoon et al., 2009). Another study found the worse functional outcomes at 3 months and 1 year after stroke in women compared to men, and these sex differences remained unchanged after adjusting for various confounding factors, including age, stroke severity, and stroke risk factors; but no sex difference was observed in the mortality rate (Smith et al., 2005). Women with AIS had greater functional impairments at 3 and 12 months after stroke, although adjusting for BI and NIHSS on admission (Wesley et al., 2010). Another Chinese study has reported that women have higher mortality, recurrence rates, and dependency rates at 3, 6, and 12 months compared to men, although the association disappeared after adjusting for age, history of diabetes, pre-stroke dependency, stroke severity, in-hospital complications, and other confounders (Zhan et al., 2013).

In the present study, women had a greater dependency rate than men at 3 and 12 months after stroke (45.39 vs. 38.33 and 33.89 vs. 26.33%, respectively), although no sex differences in mortality or recurrence rate were observed at these follow-ups. However, the sex difference in dependency rates at 3 and 12 months after stroke disappeared after adjusting for stroke subtypes, severity, and risk factors. Simultaneously, we found a significant positive association between dependency rate and stroke severity. These results indicate that sex is not the independent risk factors of dependency after stroke among elderly patients with AIS. The worse outcome among elderly women in this study is attributed to severe stroke situation on admission. Moreover, lower socioeconomic class, less social support, and social communication, which may result in indirectly less control their stroke risk factors or rehabilitate after stroke, increased depression and lower quality of life among women; these may also partially explain the worse outcomes after stroke among elderly women (Mehndiratta et al., 2015).

There are several limitations in this study. First, as all our patients were from a local neurological hospital in Tianjin, China, they cannot be considered representative of all stroke patients in China, particularly patients from rural areas. Second, we did not collect information regarding several important pre-stoke conditions, including pre-stroke dependency and time from stroke onset to hospital admission. Third, a small number of patients with AIS (1.08% at 3 months and 2.89% at 12 months) were followed up via telephone, which only allowed us to obtain information regarding death or recurrence, and not mRS score. This may have affected our evaluation of the dependency rate at 3 and 12 months after stroke.

## Conclusion

We assessed the sex differences according to stroke subtypes, severity, risk factors, and outcomes at 3 and 12 months after stroke using a large hospital-based stroke registry of elderly patients with AIS from Tianjin, China. There are significant sex differences in stroke severity, and risk factors women are more likely to have severe stroke, hypertension, dyslipidemias, and obesity. Simultaneously, more women experience the dependency after stroke, but this is resulted from severe stroke situation in women. Therefore, sex is not the independent risk factors of stroke outcome; it affected the stroke outcomes by contribution to poor stroke severity in women. Thus, we suggest that elderly female post-AIS patients are a crucial population that should be assisted with activities of daily living and controlling their risk factors for stroke.

## Author Contributions

JWu and ZA contributed to the conception and design of the work; JWu, CY, WZ, WW, CG, and SL contributed the data acquisition; JWang and JWu contributed the analysis and interpretation of data for the work; CY contributed drafting the work, JWu, ZA contributed revising the work for important intellectual content. All authors approved of the final version to be published, and agree to be accountable for all aspects of the work in ensuring that questions related to the accuracy or integrity of any part of the work are appropriately investigated and resolved.

## Conflict of Interest Statement

The research was conducted in the absence of any commercial or financial relationships that could be construed as a potential conflict of interest.

## Funding

The work reported in this article was generously supported by Grants from Tianjin Public Health Bureau (2013KG122 to Jialing Wu), (2013KG120 to Zhongping An), and Tianjin Municipal Science and Technology Commission (13JCYBJC23200 to Jialing Wu).
